# Daily Bicycle and Pedestrian Activity as an Indicator of Disaster Recovery: A Hurricane Harvey Case Study

**DOI:** 10.3390/ijerph16162836

**Published:** 2019-08-08

**Authors:** Annie Doubleday, Youngjun Choe, Scott Miles, Nicole A. Errett

**Affiliations:** 1 Department of Environmental and Occupational Health Sciences, School of Public Health, University of Washington, Seattle, WA 98195, USA; 2 Department of Industrial and Systems Engineering, University of Washington, Seattle, WA 98195, USA; 3 Department of Human Centered Design and Engineering, University of Washington, Seattle, WA 98195, USA; 4 Department of Health Services, School of Public Health, University of Washington, Seattle, WA 98195, USA

**Keywords:** wellbeing, physical activity, disaster recovery

## Abstract

Changes in levels and patterns of physical activity might be a mechanism to assess and inform disaster recovery through the lens of wellbeing. However, few studies have examined disaster impacts on physical activity or the potential for physical activity to serve as an indicator of disaster recovery. In this exploratory study, we examined daily bicycle and pedestrian counts from four public bicycle/pedestrian trails in Houston, before and after Hurricane Harvey landfall, to assess if physical activity returned to pre-Harvey levels. An interrupted time series analysis was conducted to examine the immediate impact of Harvey landfall on physical activity; *t*-tests were performed to assess if trail usage returned to pre-Harvey levels. Hurricane Harvey was found to have a significant negative impact on daily pedestrian and bicycle counts for three of the four trails. Daily pedestrian and bicycle counts were found to return to pre-Harvey or higher levels at 6 weeks post-landfall at all locations studied. We discuss the potential for further research to examine the trends, feasibility, validity, and limitations of using bicycle and pedestrian use levels as a proxy for disaster recovery and wellbeing among affected populations.

## 1. Introduction

Disasters have short and long-term impacts on physical and mental health, as well as healthcare infrastructure [1]. Several studies have examined clinical indicators of physical and mental health after disasters, including post-traumatic stress disorder, injuries, gastrointestinal infections, and diabetes-related complications [2,3,4]. Flooding-related disasters, in particular, have been shown to result in negative impacts to health [4]. However, there has been limited research on the impact of disasters on physical activity, which could act as an indicator of wellbeing. 

According to the Centers for Disease Control and Prevention, wellbeing “includes the presence of positive emotions and moods…, the absence of negative emotions..., satisfaction with life, fulfillment and positive functioning” [5]. Existing studies have largely leveraged survey methods to assess disaster-related wellbeing impacts. For instance, following the 2010–2011 Canterbury earthquakes, the Canterbury Earthquake Recovery Authority surveyed the affected individuals to assess and track wellbeing [6]. Quality of life (QOL) scores have also been used to determine the wellbeing of survivors after a disaster [7,8]. Wu et al. surveyed survivors of a 1999 Taiwanese earthquake, and found that QOL scores decreased more for the group with worse pre-existing mental health [7], and Ardalan et al. found lower QOL scores among elderly survivors of the Bam earthquake in Iran five years after the event compared to the pre-earthquake scores [8]. While these studies provide insights about the impact of disasters on wellbeing, conducting surveys to assess wellbeing following a disaster is time and resource intensive and often does not provide pre-event measurements to serve as a comparison. Thus, existing or secondary data streams that are updated continuously provide opportunities to monitor changes to wellbeing after a disaster. Further, these case studies highlight the wellbeing impacts of tsunami and earthquake events, but limited literature exists for hurricane and flood-related events, highlighting the need for additional research following meteorological disasters of differing magnitudes and impacts.

Substantial evidence has linked physical activity with improved wellbeing across different populations, and has shown sedentary activity to be associated with a reduction in overall wellbeing [9,10,11,12]. Despite known associations between physical activity and improved mental health and wellbeing [9,10,11,12,13,14,15], there is limited research on the impacts of disasters on physical activity. A few relevant studies have focused on physical activity impacts to children/adolescents or older adults and have leveraged primary data collection. For example, 8 months after Hurricane Ike, hurricane exposure and stress from recovery were associated with symptoms of post-traumatic stress and increased sedentary activity among children in Texas [16]. Another study examined the physical activity levels in children and adolescents before and after the 2011 Great East Japan earthquake. They found a decrease in physical activity after the earthquake among the children and adolescents who survived [17]. In a third study, Tsuji et al. surveyed older survivors of the same earthquake and found lower depression scores among those who participated in group exercise activities or daily walking [18]. 

In recent years, daily counts of bicycles and pedestrians on particular trails or roadways have been collected and made publicly available, e.g., in New York City and Los Angeles [19,20]. The Texas Department of Transportation consolidates daily bicycle and pedestrian count data collected by various entities in Houston and in other cities across the state [21]. Daily bicycle and pedestrian data have not been used to assess disaster impacts to physical activity, but due to such programs, these data can be rapidly and continuously accessed and provide an opportunity to assess disaster impacts to physical activity, as well as observe recovery trends. These data do not rely on self-reported information and might be able to serve as a faster and more accessible indicator of community wellbeing in disaster recovery settings.

In an effort to understand the potential to use continuously collected, and publicly available bicycle and pedestrian data to assess the physical activity impacts of disasters and monitor trends during recovery, we conducted a case study in Houston, TX, USA, following the 2017 storm, Hurricane Harvey. Hurricane Harvey made landfall on the Texas Coast on 25 August 2017, and moved slowly across southeast Texas, raining heavily in Houston and Harris County through 27 August. Harvey then slowly moved offshore, continuing to rain heavily throughout 29 and 30 August, resulting in over 40 inches of rain in some areas in Houston over this period [22]. The massive rainfall resulted in severe flooding across Houston, forcing tens of thousands of residents to evacuate [22], and resulting in closures of many bicycle and pedestrian trails for several days following landfall [23]. 

## 2. Materials and Methods 

**Figure 1:**
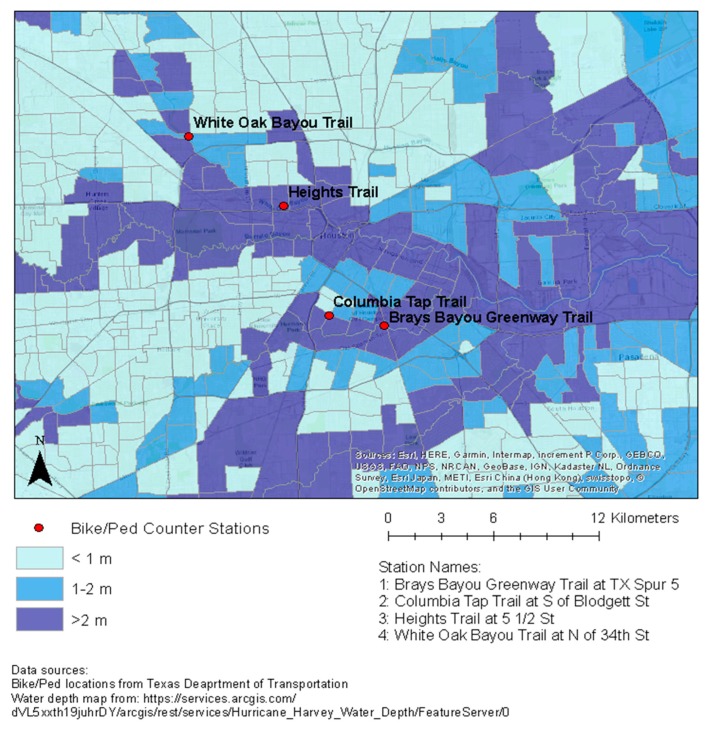
Maximum Post-Harvey flood depth (averaged across each census tract) at the four bicycle and pedestrian count stations. (The water depth layer is from here: https://services.arcgis.com/dVL5xxth19juhrDY/arcgis/rest/services/Hurricane_Harvey_Water_Depth/FeatureServer/0).

We received pedestrian and bicycle count data from the Texas Department of Transportation. The data received were at 15-min intervals from 2013 to 2018 and covered 8 counter stations located on pedestrian and bicycle trails across Houston. The coverage over the six-year period varied substantially by station within Houston, with only 4 of the 8 stations covering both the month before and after the Harvey landfall. The four stations included in the analysis were: Brays Bayou Greenway Trail, Columbia Tap Trail, Heights Trail, and White Oak Bayou Trail (Figure 1). We aggregated the count data by day and counter station to yield daily bicycle and pedestrian counts by station within Houston. We then mapped the station locations to visualize their spatial coverage and proximity, and daily counts were plotted over time by station to assess for seasonality and for a lagged effect. 

To assess the immediate impact of Hurricane Harvey on pedestrian and bicycle trail use, we analyzed the daily count data using an interrupted time series analysis (ITSA) approach. A linear regression model was fitted to the daily pedestrian and bicycle count data with a time variable to indicate pre- and post-Harvey landfall, which was defined as 25 August 2017. The pre-landfall period was defined as the month before Harvey landfall (25 July 2017–24 August 2017), and the post-landfall period was defined as the two-week period following Harvey landfall (25 August 2017–7 September 2017). The regression lines were plotted by station, separately for pedestrian and bicycle counts, to visualize the change in daily counts before and after landfall. These plots were overlaid onto the daily counts, with a LOESS (locally estimated scatterplot smoothing) smoother for visualization of the count trend over time. 

To evaluate the recovery of the pedestrian and bicycle trail use, we used Welch two-sample *t*-tests. Specifically, we assessed the statistical significance of the difference between the average daily counts in the 6 weeks before Harvey landfall and the average daily counts in the 6 weeks after Harvey landfall, excluding the two-week period immediately post-landfall, during which flooding remained widespread. The *t*-tests allowed us to formally determine whether the post-event counts returned to the pre-event level (or higher) in 6 weeks, excluding the first two weeks. During this two-week period, some of the trails in Houston were closed, impeding the trail access as a result [23]. An employee at the Houston Parks Board informed us of the degree of damage and extent of closures for several trails that were included in our analysis. We used this information to define the post-landfall period in the recovery analysis. Recovery was categorized by a return to pre-event counts (no significant difference in counts) or an increase in counts in the 6-week period following landfall, compared to the 6-week period prior to landfall. The average daily counts in the 6 weeks before and after the Harvey landfall were then plotted by station and mode of transportation (i.e., bicycle or pedestrian use), for comparison. All analyses were performed using R 3.5.2 (R Core Team, Vienna, Austria). Figure 1 was prepared using ArcMap 10.5.1 (Esri, Redlands, CA, USA).

## 3. Results

Figure 1 displays the location of the four stations in our analysis, and includes the maximum post-Harvey flood depth, averaged across each census tract.

**Figure 2:**
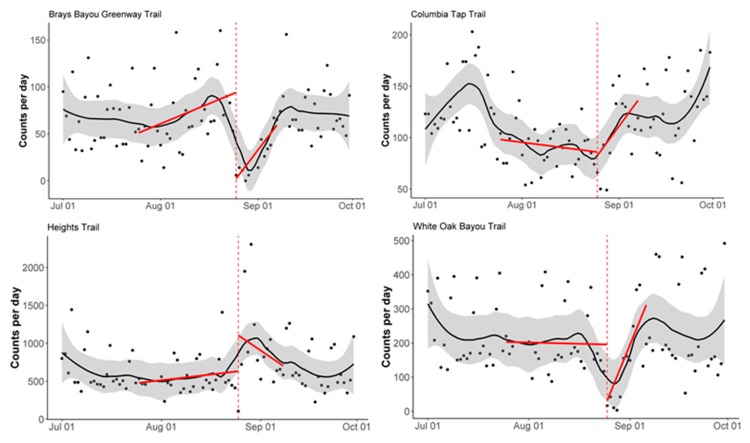
Interrupted time-series analysis of bicycle counts by station. The vertical dashed red line corresponds to Harvey landfall (25 August 2017). The solid red lines correspond to the interrupted time-series regression lines. The solid black line corresponds to the LOESS smoother and the grey shading corresponds to a 30% smoothing span around the LOESS smoother line.

**Figure 3:**
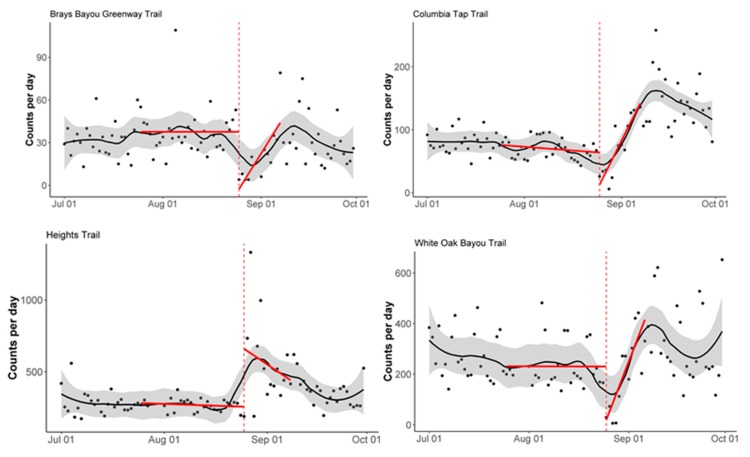
Interrupted time-series analysis of the pedestrian counts by station. The vertical dashed red line corresponds to the Harvey landfall (25 August 2017). The solid red lines correspond to the interrupted time-series regression lines. The solid black line corresponds to the LOESS smoother, and the grey shading corresponds to a 30% smoothing span around the LOESS smoother line.

Figure 2 and Figure 3 display the results of the interrupted time-series analysis. All stations except the Heights Trail station saw a significant decrease (*p* < 0.05) in bicycle counts immediately following Harvey landfall (Figure 2). Both the Columbia Tap Trail station and the White Oak Bayou Trail station also saw a significant change in slope (*p* < 0.05), when comparing the month before landfall to the two-week period following landfall. Both stations saw a decrease in counts at the time of landfall, followed by an increase in counts, yielding the significantly steeper slopes in the post-landfall period.

All stations except the Heights Trail station saw a significant decrease (*p* < 0.05) in pedestrian counts immediately following Harvey landfall (Figure 3). The same three stations also saw a significant change in slope (*p* < 0.05), when comparing the month before landfall to the two-week period following landfall. All three stations saw a decrease in counts at the time of landfall, followed by an increase in counts, yielding the significantly steeper slopes in the post-landfall period.

Table 1 shows the results of the recovery analysis using Welch two-sample *t*-tests. All four stations saw an increase in the mean daily counts in the 6 weeks after landfall (excluding the two-week period after landfall), as compared to the 6 weeks prior to landfall for both pedestrian and bicycle counts, except for pedestrian counts at Brays Bayou Greenway Trail.

**Figure 4:**
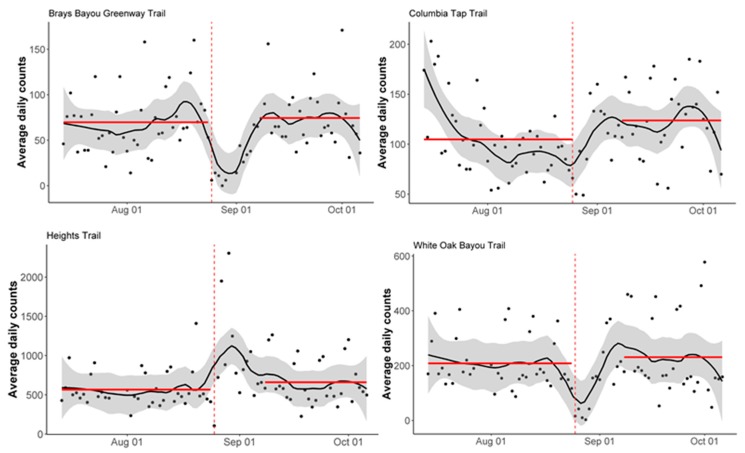
Averages of the daily bicycle counts, by station, in the 6 weeks before and after landfall, excluding the two-week period after landfall. The vertical dashed red line corresponds to the Harvey landfall (8/25/2017). The red lines correspond to the average daily bicycle counts. The solid black line corresponds to the LOESS smoother, and the grey shading corresponds to a 30% smoothing span around the LOESS smoother line.

**Figure 5:**
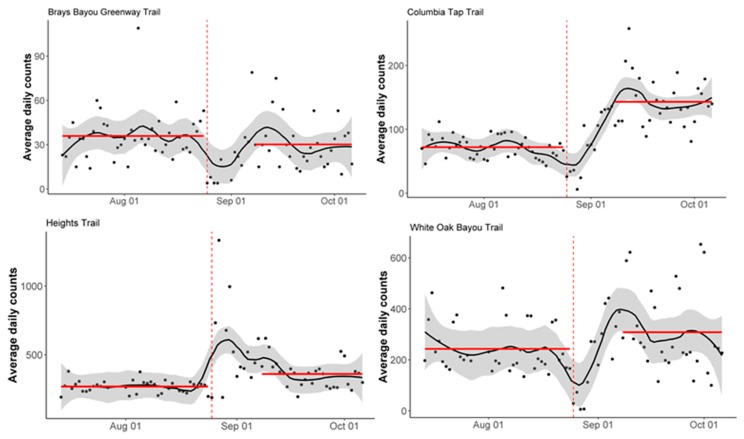
Averages of daily pedestrian counts, by station, in the 6 weeks before and after landfall, excluding the two-week period after landfall. The vertical dashed red line corresponds to the Harvey landfall (25 August 2017). The red lines correspond to the average daily pedestrian counts. The solid black line corresponds to the LOESS smoother, and the grey shading corresponds to a 30% smoothing span around the LOESS smoother line.

Figure 4 and Figure 5 show the average daily bicycle and pedestrian counts in the six weeks before and after Harvey landfall, excluding the two-week period after landfall. All trails experienced an increase in average daily counts or return to pre-landfall average daily counts in the post-landfall period (with the exception of the statistically insignificant decrease in pedestrian counts for the Brays Bayou Greenway Trail).

## 4. Discussion

To the best of our knowledge, this exploratory study is the first of its kind to compare pre-event and post-event bicycle and pedestrian activity trends, and it is the first to use publicly available bike and pedestrian activity data to do so. Most existing studies examine physical activity patterns during a much longer post-event period and do not compare the pre- and post-event physical activity levels [16,17]. As such, this work provides unique insights into both the immediate disaster impacts on physical activity, as well as the potential for such publicly available data to be used to monitor recovery progress following extreme events. 

The results from the interrupted time-series analysis indicate a significant decrease in daily counts in the time-period immediately following Harvey landfall at nearly all stations included in the study. This might be explained by the fact that the corresponding trails in our analysis were underwater for several days following landfall. While this might be seen as an inevitable confounding factor in the analysis, it provides insight about the potential for this publicly accessible data to be used as a tool for post-event reconnaissance. Given these data are continuously tracked and could be made available in near real-time, they have the potential to be an important data source for emergency managers and decision makers if it is used as a spatial marker of infrastructure damage or as an indicator of wellbeing. Additional research is necessary to correlate the observed impacts with other traditional indicators of recovery (e.g., critical infrastructure restoration) and wellbeing impacts (e.g., measured through QOL scores or other validated instruments) to determine the utility of bicycle and pedestrian trail data as a near real-time indicator for recovery and wellbeing, respectively.

The results from the recovery analysis indicate an increase in the average daily counts in the 6-week period after landfall (excluding the initial two-week period) compared to the 6-week period prior to landfall among most stations, although only some stations saw a statistically significant increase. This result differs from other studies, which have found no evidence for return to normal or an increase in physical activity levels after a disaster [16,17]. Okazaki et al. [17] reported accelerometer-determined daily steps in adolescents each year for three years following a 2011 earthquake, and found a significantly lower step count at three years compared to one year following the earthquake. Similarly, Lai et al. [16] reported sedentary activity in children 8 months after Hurricane Ike. Both of these studies followed children’s physical activity for long periods after disasters, and did not find evidence for return to normal levels of physical activity. These findings are not consistent with our results in the recovery analysis examining the impact of Harvey landfall on physical activity up to six weeks after landfall. We examined a significantly shorter time period than those studied in Okazaki et al. [17] and Lai et al. [16], and as such, it is possible the trends observed here might not continue in the months and years following Harvey landfall. Examination of longer time-periods may yield different conclusions about post-event physical activity trends on bike and pedestrian trails. 

The lack of individual user data impedes the ability to attribute the change in activity to changes in use by any individual. It is possible that the trail users chose alternate locations to walk or bike in the immediate aftermath of the event, or that different people accessed the trails for different reasons before and after the event. For instance, as many as 1 million cars were damaged following Hurricane Harvey [24] and it is possible that increased trail usage following the event could represent an increase in temporary or permanent active transportation users. As such, additional research is necessary to understand disaster-related impacts on individual-level physical activity patterns and their determinants. Prospective research should use data sources that provide individual metrics of physical activity (e.g., through personal health monitoring devices) and surveys to assess the use of alternative modes of activity (e.g., gym usage) and determinants of pre- and post-event physical activity. 

While physical activity has been associated with wellbeing in non-disaster settings [10,11,12], we did not explicitly correlate bicycle and pedestrian activity with markers of wellbeing. Additionally, as noted above, the lack of individual user data further impedes our ability to attribute changes in trail use with disaster-related changes in individual wellbeing. Additional data collection, including through surveys (e.g., using the Centers for Disease Control and Prevention’s Community Assessment for Public Health Emergency Response (CASPER) epidemiologic technique [25]) and interviews, might provide more insights on the role and association of physical activity and physical activity-promoting infrastructure on wellbeing in a post-event setting. Prior to use of activity data as an indicator of post-event wellbeing, research should explore the association, if any, of physical activity changes with post-event wellbeing indicators. As noted above, there are many potential reasons for changes in physical activity following a disaster that might confound the use of activity data as an indicator of post-event wellbeing. 

Finally, communities with bike and pedestrian infrastructure might be systematically different from those without access to such community amenities, and thus our results might not be generalizable. A study associating zoning code requirements with physical activity data from the 2011 Behavioral Risk Factor Surveillance System found 58% higher odds of biking and 52% higher odds of vigorous biking, as well as 5% (non-significant) higher odds of pedestrian activity, among communities with zoning-based bike and pedestrian infrastructure [26]. Moreover, communities with zoning provisions for bike/pedestrian infrastructure have reported significant, albeit small, increases in rates of active transportation to work [27]. As such, it is possible that members of communities with bicycle and pedestrian infrastructure are more likely to be physically active even before disaster events. Given this, bicycle and pedestrian activity data should be used and interpreted with caution, especially in post-event and recovery decision-making. These data might disproportionately detect changes in those who are physically active prior to an event, and miss important impacts experienced by communities who are not physically active, or whose physical activity is not well-represented by bicycle and pedestrian activity data.

## 5. Conclusions

This exploratory analysis is the first of its kind to compare physical activity trends before and after a flood-related disaster using publicly available bicycle and pedestrian activity data. This analysis provides important insights regarding physical activity impacts of a disaster and explores the potential for using bicycle and pedestrian activity data as an indicator of disaster recovery. This research found that bicycle and pedestrian activity decreased immediately following Hurricane Harvey landfall, with a return to pre-event levels in 6 weeks post-event. However, this exploratory analysis was limited by the number of counter stations, length of the analysis period, lack of additional variables, and possible lack of applicability in other communities and following other disaster events. It may not be feasible to use bicycle and pedestrian activity data after an event with more extensive infrastructure damage, or in communities without bike and pedestrian infrastructure and counters or without high levels of infrastructure usage. Additional research is necessary to explore the potential to use bicycle and pedestrian activity data in other communities and following other types of disaster events with different levels of impacts, as well as to assess longer-term physical activity patterns. Finally, external factors that may impact post-event physical activity trends are not accounted for here, including level of damage and flood depth by location, temperature, humidity, and air quality. As such, additional research should be conducted to explore physical activity levels and their environmental determinants in post-event settings, including secondary and concurrent hazards. Further research is necessary to understand the feasibility, validity, and limitations of using individual physical activity data for tracking recovery and wellbeing after disasters. This includes a need for larger prospective studies with individual-level data and those following disasters caused by different hazards, with different levels of damage. 

## Figures and Tables

**Table 1:** Results of the *t*-test for counts 6 weeks before and after Harvey landfall.

Station	Pedestrian			Bicycle		
	Pre-Harvey Average ^1^	Post-Harvey Average ^2^	Difference in Means	Pre-Harvey Average ^1^	Post-Harvey Average ^2^	Difference in Means
Brays Bayou Greenway Trail	36	30	*p* = 0.14	70	74	*p* = 0.56
Columbia Tap Trail	72	143	*p* < 0.001	105	124	*p* = 0.04
Heights Trail	268	360	*p* < 0.001	566	660	*p* = 0.15
White Oak Bayou Trail	243	308	*p* = 0.06	208	231	*p* = 0.48

**^1^** The pre-Harvey average is the average of daily counts in the 6 weeks prior to Harvey landfall. ^2^ The post-Harvey average is the average of daily counts in the 6 weeks following Harvey landfall, excluding the two-week period immediately following landfall.
